# Assessing the role of advanced artificial intelligence as a tool in multidisciplinary tumor board decision-making for primary head and neck cancer cases

**DOI:** 10.3389/fonc.2024.1353031

**Published:** 2024-05-24

**Authors:** Benedikt Schmidl, Tobias Hütten, Steffi Pigorsch, Fabian Stögbauer, Cosima C. Hoch, Timon Hussain, Barbara Wollenberg, Markus Wirth

**Affiliations:** ^1^ Department of Otolaryngology Head and Neck Surgery, Technical University Munich, Munich, Germany; ^2^ Department of RadioOncology, Technical University Munich, Munich, Germany; ^3^ Institute of Pathology, Technical University Munich, Munich, Germany

**Keywords:** HNSCC, multidisciplinary tumor board, radiotherapy, artificial intelligence, ChatGPT

## Abstract

**Background:**

Head and neck squamous cell carcinoma (HNSCC) is a complex malignancy that requires a multidisciplinary approach in clinical practice, especially in tumor board discussions. In recent years, artificial intelligence has emerged as a tool to assist healthcare professionals in making informed decisions. This study investigates the application of ChatGPT 3.5 and ChatGPT 4.0, natural language processing models, in tumor board decision-making.

**Methods:**

We conducted a pilot study in October 2023 on 20 consecutive head and neck cancer patients discussed in our multidisciplinary tumor board (MDT). Patients with a primary diagnosis of head and neck cancer were included. The MDT and ChatGPT 3.5 and ChatGPT 4.0 recommendations for each patient were compared by two independent reviewers and the number of therapy options, the clinical recommendation, the explanation and the summarization were graded.

**Results:**

In this study, ChatGPT 3.5 provided mostly general answers for surgery, chemotherapy, and radiation therapy. For clinical recommendation, explanation and summarization ChatGPT 3.5 and 4.0 scored well, but demonstrated to be mostly an assisting tool, suggesting significantly more therapy options than our MDT, while some of the recommended treatment modalities like primary immunotherapy are not part of the current treatment guidelines.

**Conclusions:**

This research demonstrates that advanced AI models at the moment can merely assist in the MDT setting, since the current versions list common therapy options, but sometimes recommend incorrect treatment options and in the case of ChatGPT 3.5 lack information on the source material.

## 1 Introduction

The incidence of cancer worldwide is more than 24.5 million cases, resulting in 9.6 million deaths per year (1). Head and neck squamous cell carcinoma (HNSCC), ranking as the seventh most common type of cancer, poses unique challenges due to the complexity of the anatomical region and heterogeneous nature of disease. Consequently, the most common approach is the discussion of oncological cases in a multidisciplinary tumor board (MDT). MDTs are designed to improve patient outcomes through an individualized and collaborative strategy (2). They bring together a diverse team of medical professionals, including medical and surgical oncologists, radiation oncologists, pathologists, and radiologists to discuss the treatment of cancer patients (1, 3). MDTs provide a comprehensive perspective on each case, tailoring treatment plans to individual needs, especially through molecular tumor boards for difficult-to-treat cases (3, 4). Despite their benefits, MDTs face obstacles such as costs, responsibilities, geographic barriers, and treatment delays (2–4).

In parallel, the field of artificial intelligence (AI), especially deep learning (DL) and natural language processing (NLP), has proven significant advancement (5). Models like Generative Pre-trained Transformer (GPT), developed by OpenAI (San Francisco, California) in 2018, have surpassed earlier AI tools in resources and capabilities (5). These AI models have already shown potential in oncology, as exemplified by IBM Watson, and are now being explored for their utility in assisting clinical decision-making (6).

While AI at the moment cannot replace the expertise of healthcare professionals, it may aid in decision-making by efficiently accessing and presenting relevant information. Our group’s previous work demonstrated that ChatGPT accurately answered 65% of ENT tumor related practice questions for the German otolaryngology board certification (7). However, the comparative effectiveness of ChatGPT 3.5 and 4.0 in assisting MDTs for primary cases of head and neck cancer remains to be elucidated.

This study aims to assess the potential of ChatGPT 3.5 and 4.0 in providing suitable recommendations for patients diagnosed with primary head and neck cancer, comparing its performance with traditional MDTs.

## 2 Materials and methods

### 2.1 Patient cohort

The inclusion criteria comprised a confirmed diagnosis of head and neck cancer and the absence of distant metastasis. Excluded from consideration were cases involving recurrent situations. Tumor characteristics and the age of the ten consecutive patients before treatment were obtained from the MDT. In this study, a total of 20 patients were included. These cases were discussed in the MDT of October 2023 in the Department of Otorhinolaryngology/Head and Neck Surgery, Klinikum rechts der Isar, Technical University of Munich. The age of patients in this study ranged from 52 to 74 years (Median age 61.5, Average age 63.8). The data were anonymized before being provided to the investigators, ensuring that patient identification was not possible. Subsequently, the extracted information (**Table 1**) was entered into the ChatGPT 3.5 or 4.0 chat bot. The resulting answers were then copied and categorized.

**Table 1:** Overview of the patient cohort and the information entered into ChatGPT.

Patient_ID	Age	cTx	cNx	cMx	Localization	p16 status	Smoking status	Comorbidities	KPS	Specific recommendation of the MDT
1	74	4a	0	0	Transglottic larynx left	NA	No more, 25 py	aHTN	100%	Surgery + Adj. RTx vs RCTx
2	62	2	2b	0	Hypopharynx right	NA	Active, 40 py	pAVK, DM type II	60%	Surgery + Adj. RCTx
3	60	3	0	0	Oropharynx right	–	Active, 25py	aHTN	100%	Surgery + Adj. RTx
4	61	2	1	0	Hypopharynx left	NA	Rarely	Chronic renal insufficiency	50%	Surgery + Adj. RTx
5	61	3	1	0	Oropharynx left	+	Never	aHTN	100%	Surgery + Adj. RCTx
6	52	2	1	0	Oropharynx left	+	Never	aHTN	100%	Surgery + Adj. RCTx
7	63	1b	0	0	Glottic larynx	NA	Active, 40 py	Alcohol abuse	80%	Surgery + Clinical Controls
8	73	2	0	0	Oropharynx right	+	Never	DVT 2015	100%	Surgery + Adj. RTx
9	70	3	0	0	Right nasal cavity	NA	Never	CLL, aHTN	100%	Surgery + Adj. RTx
10	57	4a	1	0	Supraglottic larynx to subglottic with thoracic extension and into the trachea.	NA	Never	h.o. pneumonia, COPD	60%	RCTx
11	53	3	1	0	Oropharynx right	+	Never	aHTN	100%	Surgery + Adj. RTx
12	71	1b	0	0	Glottic both sides	NA	Active, 20 py	DM type II	60%	Surgery
13	61	2	2c	0	Oropharynx right	+	Never	aHTN	90%	Surgery + Adj. RTx
14	59	4a	1	0	Oropharynx both sides	–	Active, 80 py	Chronic renal insufficiency	50%	RCTx
15	73	4a	0	0	Transglottic	NA	Active, 60 py	pAVK, Chronic renal insufficiency	60%	Surgery + Adj. RTx
16	74	3	0	0	Hypopharynx right	NA	Active, 40 py	aHTN, Chronic renal insufficiency	100%	Surgery + Adj. RTx
17	56	2	2c	0	Hypopharynx right	NA	Never	aHTN	60%	Surgery + Adj. RTx
18	60	3	2b	0	Glottic left	+	Active, 30 py	None	80%	Surgery + Adj. RTx
19	66	4a	1	0	Glottic	NA	Active, 40 py	aHTN, Chronic renal insufficiency	60%	Surgery + Adj. RTx
20	70	3	1	0	Oropharynx left	+	Never	None	100%	Surgery + Adj. RTx

In addition, the result of the MDT presentation is also depicted in this table, even though ChatGPT was not able to access this information. NA, not available; DVT, deep vein thrombosis; aHTN, arterial hypertension; DM, diabetes mellitus; TLE, Total Laryngectomy; sND, selective neck dissection; Adj, adjuvant; RCTx, radiochemotherapy; KPS, Karnofsky Performance Index; RT, radiotherapy.

+ = positive, - = negative.

Lowercase letters are part of the TNM classification.

Ethical approval was waived by the ethics committee of the Technical University of Munich due to the retrospective nature of this study.

### 2.2 Artificial intelligence/ChatGPT

ChatGPT is a publicly accessible artificial intelligence chatbot, that is based on a transformer-based language model. It can produce text that is similar to human language. A special characteristic is that it is only trained on data up to 2021. The user can input questions via a website, and ChatGPT analyzes the contextual relationships among words in the query. The training data for ChatGPT stem from various open-access internet sources, including websites, articles, and books, up until the year 2021 (4–8) GPT model 3.5 and ChatGPT 4.0. were used, available publicly in October 2023.

### 2.3 ChatGPT prompt format and data evaluation

A standardized prompt format was used for inputting patient information into ChatGPT, mimicking the setting of an individual patient presentation in MDTs. The prompt used for this study was the standardized way of presenting a case at the MDT to allow a direct comparison of the results to the recommendations of the MDT. Before this prompt was used, four different prompts were tested to validate the results, since a lack of information leads to insufficient and vague answers. The other three prompts are depicted in **Supplementary Figure 1**. The prompt was as follows: “A (XX) year old patient with a cT (XX) cN (XX) squamous cell carcinoma of the (XX), the patient (XX) smokes, (XX) and has the following secondary diseases (XX) with a Karnofsky Index of (XX). The patient is presented in an interdisciplinary tumor board. What treatment options are available and which option do you think leads to the best prognosis?”. This setup replicates an oncologist/head and neck surgeon interaction with ChatGPT. An example would be: “A 74-year-old patient with a cT4a cN0 squamous cell carcinoma of the left transglottic larynx, the patient no longer smokes, cum. 25 pack/years and has the following secondary diseases: arterial hypertension with a Karnofsky Index of 100%. The patient is presented in an interdisciplinary tumor board pre-therapeutically. What treatment options are available and which option do you think leads to the best prognosis?” Following each response, no further dialogue was initiated; the ChatGPT history was cleared, and the subsequent question was posed. All the prompts can be found in the **Supplementary Data**. A new ChatGPT session was started for each prompt to avoid any sort of training or influence by prior answers. Each answer by ChatGPT was collected by B.S. and reviewed by B.S. and T.H. independently. For each treatment modality that was mentioned one point was given to ChatGPT. Additionally, the grading scales for Summarization, Clinical Recommendation and Explanation as already used by Sorin et al., 2023 (9) were used to rate the answers by ChatGPT 3.5 and 4.0. The grading scales are depicted in the **Supplementary Figure 1**. The scoring transitioned from a baseline of zero points to a maximum of five points in these grading scales. The whole treatment history was considered. The inter-rater reliability was measured using Cohen’s kappa coefficient.

## 3 Results

**Figure 1:**
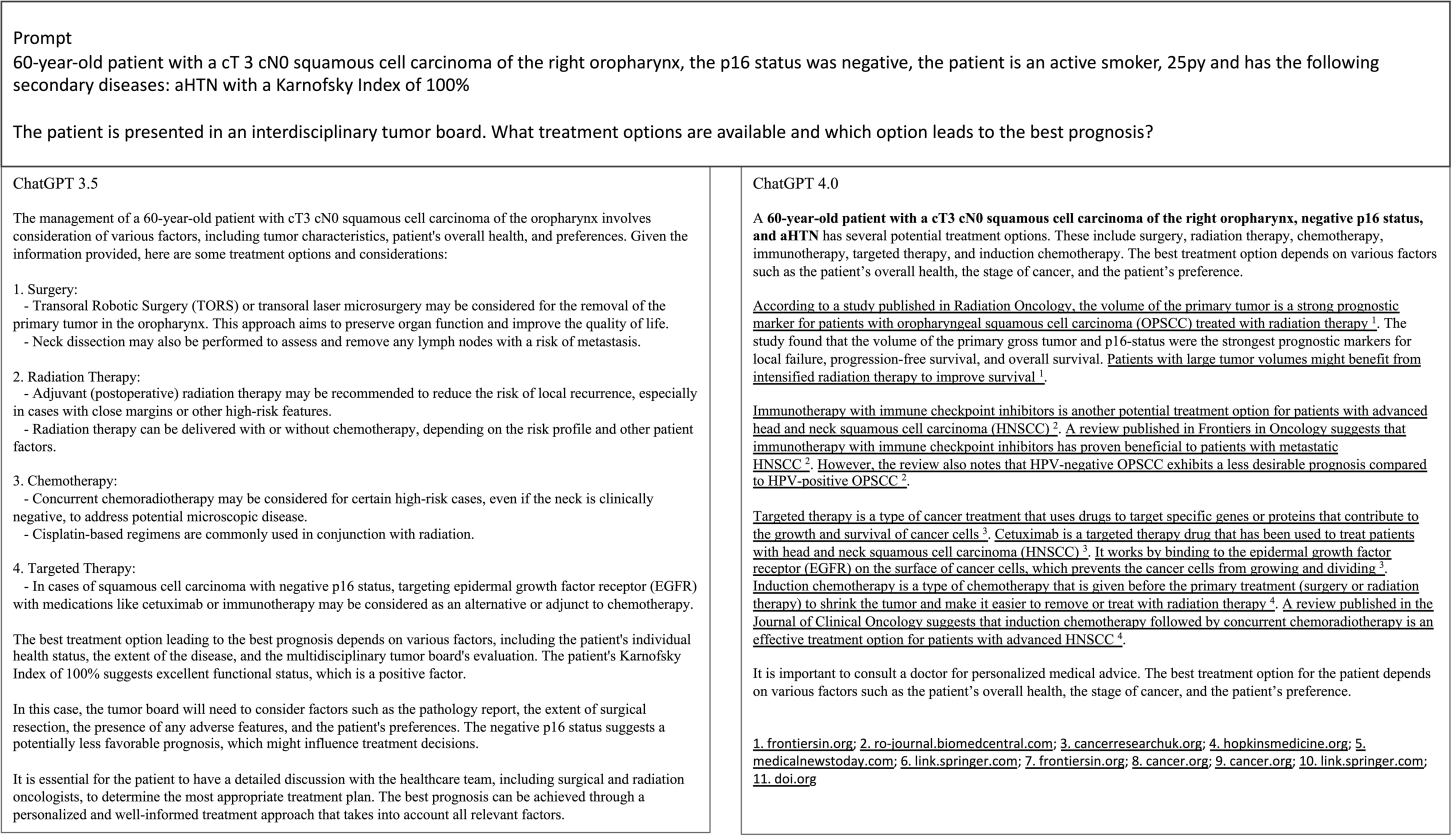
Depiction of an exemplary prompt and responses by ChatGPT 4.0 and 3.5. The answers were copied from the response page.

The most common response from ChatGPT 3.5 involved the primary treatment modalities available for potential use in a primary or adjuvant setting, including surgery, radiotherapy, and chemotherapy. An exemplary prompt and the answers generated by ChatGPT 3.5 and ChatGPT 4.0 are depicted in **Figure 1**. When comparing the MDT recommendations with those of ChatGPT 3.5, two independent reviewers reached an agreement measured by Cohen’s κ of 1 for the number of therapy options, of Cohen’s κ of 0.459 for summarization of text, of 0.44 for clinical recommendation, and 0.579 for explanation on the decision made. ChatGPT 4.0 reached a Cohen’s κ of 1 for the number of therapy options, of Cohen’s κ of 0.612 for summarization of text, of 0.455 for clinical recommendation, and 0.452 for explanation on the decision made.

In absolute numbers ChatGPT 3.5 suggested on average a significantly higher number of treatment options for the patients in this study (4.85), compared to the MDT (**Table 2**). A summary and depiction of the results is presented in **Table 2**. Immunotherapy was recommended for 60% of the patients by ChatGPT 3.5 in this study, even in primary early stage HNSCC, without regard to the PD-L1 status or combined positive score (CPS). Neoadjuvant trials were not recommended by ChatGPT 3.5 or ChatGPT 4.0 as well as other strategies like HPV vaccination. The age of the patients was emphasized by ChatGPT 3.5 with a potential inability to endure surgery or chemotherapy. Surgery was recommended by ChatGPT 3.5 for all patients in this study (100%), also reaching the highest concordance with the MDT (18 out of 20 patients, 90%). Surgery was also recommended in two cases that explicitly had a quite advanced tumor infiltration of the supraglottic structures and into the trachea or on multiple subsites, where the MDT suggested a definitive radiochemotherapy. In these special cases a second thread in ChatGPT 3.5 was opened and it was specifically asked which therapy offers the highest quality of life. Surgery was still the first suggestion of ChatGPT 3.5, but it was mentioned that infiltration into the trachea and “other factors” may lead to surgery not being the sole therapy for this patient. ChatGPT 3.5 in general seemed to be careful not to recommend a single treatment modality and tried to balance the importance of different therapy options. On the other hand, some therapy options, such as adjuvant radiochemotherapy were not mentioned by ChatGPT. ChatGPT 3.5 and 4.0 were able to name different surgical approaches for some cases.

**Table 2:** Overview of the results and treatment options of ChatGPT 3.5 compared to the MDT.

	Rec. by MDT	Rec. by ChatGPT 3.5	# of therapy options by ChatGPT 3.5		Summarization Scale		Clinical Recommendation Scale		Explanation Scale	
Patient			Rev 1	Rev 2	Rev 1	Rev 2	Rev 1	Rev 2	Rev 1	Rev 2
1	Surgery + Adj. RTx vs RCTx	Surgery + Adj. RTx	5	5	4	5	3	5	5	5
2	Surgery + Adj. RCTx	Surgery + Adj. RTx	4	4	4	4	5	5	4	4
3	Surgery + Adj. RTx	Surgery + Adj. RTx	6	6	5	5	5	5	4	4
4	Surgery + Adj. RTx	Surgery + Adj. RTx	5	5	5	5	5	5	5	5
5	Surgery + Adj. RCTx	RCTx vs Surgery + Adj. RTx	4	4	4	4	4	5	4	4
6	Surgery + Adj. RCTx	Surgery + Adj. RCTx	4	4	4	4	4	5	4	4
7	Surgery + Clinical Controls	Surgery vs RTx	5	5	4	4	5	5	4	4
8	Surgery + Adj. RTx	Surgery + Adj. RTx	6	6	4	5	4	5	4	4
9	Surgery + Adj. RTx	Surgery + Adj. RTx	6	6	5	4	5	5	4	4
10	RCTx	Surgery + Adj. RTx	4	4	4	4	2	2	3	4
11	Surgery + Adj. RTx	Surgery + Adj. RTx	4	4	5	5	4	4	4	5
12	Surgery	Surgery	5	5	5	3	3	4	5	5
13	Surgery + Adj. RTx	Surgery + Adj. RTx	4	4	5	5	4	4	5	5
14	RCTx	Surgery + Adj. RTx	6	6	4	4	5	5	5	5
15	Surgery + Adj. RTx	Surgery + Adj. RTx	4	4	5	5	4	4	4	4
16	Surgery + Adj. RTx	Surgery + Adj. RTx	5	5	4	5	4	5	4	4
17	Surgery + Adj. RTx	Surgery + Adj. RTx	6	6	4	4	4	5	4	4
18	Surgery + Adj. RTx	Surgery + Adj. RTx	5	5	5	5	4	4	5	4
19	Surgery + Adj. RTx	Surgery + Adj. RTx	5	5	4	3	4	4	5	4
20	Surgery + Adj. RTx	Surgery + Adj. RTx	4	4	5	5	4	4	4	4
	Cohen’s κ		1		0.459		0.44		0.579	
	P value		0.00007		0.0151		0.0037		0.00439	

The answers of ChatGPT were evaluated by two independent reviewers in the categories summarization of text, clinical recommendation, and explanation on the decision made. The agreement of the Reviewers was calculated with Cohen’s κ. Adj, adjuvant; Def, definitive; RCTx, radiochemotherapy; RTx, radiotherapy.

**Figure 2:**
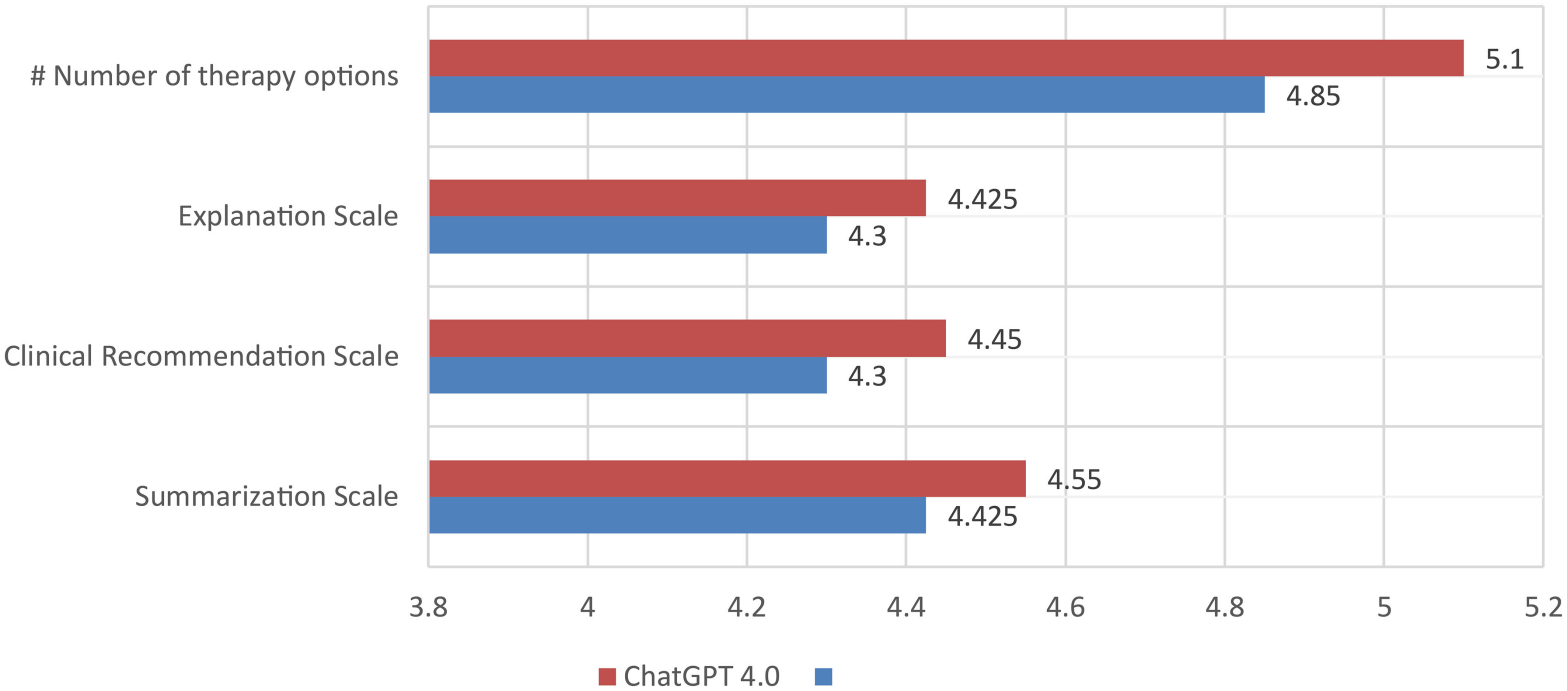
Rating of the performance of ChatGPT 4.0 and 3.5 by comparison of the number of treatment options per case and the grading of summarization of text, clinical recommendation, and explanation on the decision made by two independent reviewers. Each bar is the average of the two independent reviewers grading. # = number.

ChatGPT 4.0 suggested on average a higher number of treatment options than ChatGPT 3.5 for the patients in this study (5.1), significantly more than the MDT, which in our institution mostly recommends a single therapy, or a maximum of two therapies for each case (**Table 3**; **Figure 2**). A summary and depiction of the results is presented in **Table 3**. When analyzing the scales of summarization, clinical recommendation, and explanation, we see that ChatGPT 4.0 achieves better grades for summarization, explanation and clinical recommendation from both Reviewer 1 and 2 (**Figure 2**). Additionally, ChatGPT 4.0 is able to reference its sources, in some cases it even mentions recent clinical studies. On the other hand, ChatGPT 4.0 also refrained from giving precise recommendations, and mentioned that it is not meant to give medical advice or replace the opinion of a medical doctor. ChatGPT 4.0 recommended surgery for 18 out of 20 patients (90%), which is in concordance with the decisions of the tumor board. Two patients with extensive tumor spread, which the MDT deemed candidates for radiochemotherapy, were allocated for definitive radiochemotherapy. These patients would have received surgery according to ChatGPT 3.5, even though the extent of the tumor growth limits surgical resection, and this therapy was therefore not recommended by the MDT.

**Table 3:** Overview of the results and treatment options of ChatGPT 4.0 compared to the MDT.

	Rec. by MDT	Rec. by ChatGPT 4.0	# of therapy options by ChatGPT 4.0		Summarization Scale		Clinical Recommendation Scale		Explanation Scale	
Patient			Rev 1	Rev 2	Rev 1	Rev 2	Rev 1	Rev 2	Rev 1	Rev 2
1	Surgery + Adj. RTx vs RCTx	Surgery + Adj. RTx	6	6	5	5	4	5	5	5
2	Surgery + Adj. RCTx	Surgery + Adj. RTx	4	4	4	5	5	5	5	5
3	Surgery + Adj. RTx	Surgery + Adj. RTx	5	5	5	5	5	5	4	4
4	Surgery + Adj. RTx	Surgery + Adj. RTx	6	6	4	4	5	5	4	4
5	Surgery + Adj. RCTx	RCTx vs Surgery + Adj. RTx	6	6	4	5	4	5	5	5
6	Surgery + Adj. RCTx	Surgery + Adj. RCTx	6	6	4	4	5	5	4	4
7	Surgery + Clinical Controls	Surgery vs RTx	4	4	4	4	5	5	5	4
8	Surgery + Adj. RTx	Surgery + Adj. RTx	6	6	5	5	5	5	3	5
9	Surgery + Adj. RTx	Surgery + Adj. RTx	6	6	5	5	5	5	4	4
10	RCTx	RCTx	4	4	5	5	4	4	5	5
11	Surgery + Adj. RTx	Surgery + Adj. RTx	5	5	5	5	4	5	4	3
12	Surgery	Surgery	4	4	3	3	3	4	4	5
13	Surgery + Adj. RTx	Surgery + Adj. RTx	5	5	4	4	4	4	4	4
14	RCTx	RCTx	6	6	5	5	4	4	4	4
15	Surgery + Adj. RTx	Surgery + Adj. RTx	5	5	4	4	4	4	4	5
16	Surgery + Adj. RTx	Surgery + Adj. RTx	4	4	5	5	4	4	5	5
17	Surgery + Adj. RTx	Surgery + Adj. RTx	5	5	5	5	4	4	5	4
18	Surgery + Adj. RTx	Surgery + Adj. RTx	4	4	5	5	4	4	4	4
19	Surgery + Adj. RTx	Surgery + Adj. RTx	6	6	5	4	4	5	5	5
20	Surgery + Adj. RTx	Surgery + Adj. RTx	5	5	5	4	4	5	5	5
	Cohen’s κ		1		0.612		0.455		0.452	
	P value		0.00007		0.00173		0.0154		0.0209	

The answers of ChatGPT were evaluated by two independent reviewers in the categories summarization of text, clinical recommendation, and explanation on the decision made. The agreement of the Reviewers was calculated with Cohen’s κ. Adj, adjuvant; Def, definitive; RCTx, radiochemotherapy; RTx, radiotherapy.

## 4 Discussion

This study represents the first examination of ChatGPT 3.5 and 4.0, an open AI, as an auxiliary tool for MDTs engaged in discussions about patients with a primary diagnosis of HNSCC. The recommendations of ChatGPT 3.5 and 4.0 were compared. NLP, a subset of AI focusing on the analysis of human language, has found applications across various medical specialties, including breast cancer research, rheumatology, medical education, medical exams, and many more (8, 10–14). The diagnostic accuracy in complex medical cases was tested in a recent publication, showing that ChatGPT accurately identifies the correct diagnosis in nearly 40% of cases as its primary suggestion (15). These findings, and ChatGPT’s ability to provide logical and contextually appropriate responses to diverse text questions through the application of advanced language modeling techniques and extensive access to large and diverse datasets (10, 11) led to this study.

MDTs deal with a lot of data when reviewing a patient’s case with the goal of tailoring general treatment plans to the patient’s individual needs and therefore improving care (2). Organizing and processing data is one of the strengths of ChatGPT, which is the reason it may become an auxiliary tool for the MDT in the future. In this study ChatGPT 3.5 and 4.0 achieved good grades for clinical recommendation, explanation and summarization in this study. ChatGPT 3.5 and 4.0 listed the different treatment options in a very efficient way, highlighting its potential to enhance efficiency in clinical workflows. A recommendation for immunotherapy as a therapy option was given to 60% of the patients in this study. This rate was significantly higher (600%) than the rate of the MDT. There the recommendation was only given in a very advanced case where the tumor was already unresectable. In Germany and other countries, immunotherapy is currently limited to patients with recurrent/metastatic HNSCC, with the two antibodies that were approved by the US Food and Drug Administration (FDA), nivolumab and pembrolizumab, widely used (16–18). The recommendation of immunotherapy in the primary cases of HNSCC in this study are currently not concordant with the FDA approval and treatment guidelines. Another often recommended treatment option of ChatGPT was the definitive radiotherapy, regardless of the tumor localization. This topic is widely discussed in oropharyngeal carcinoma with primary radiotherapy and/or chemoradiotherapy still widely used, especially in settings where transoral surgery is not feasible (19, 20) as well as in hypopharyngeal carcinoma, which is often asymptomatic for a long time until a surgical therapy is not possible anymore (19). The concordance with the MDT in terms of surgical therapy was 90% (9 out of 10 patients). Surgery was recommended by ChatGPT 3.5 for all patients in this study, even for one patient with a very advanced, unresectable tumor, where the MDT suggested a definitive radiochemotherapy or immunotherapy. When ChatGPT 3.5 was in this one case asked for a therapy option with the highest quality of life, surgery was still the first therapy, but the problem of potential surgical limitations was at least addressed. ChatGPT 4.0 recommended a definitive radiochemotherapy for this patient and was therefore superior in terms of concordance with the MDT. ChatGPT 3.5 reached high scores in terms of explanation, clinical recommendation and summarization by both reviewers in this study. The explanation grade was graded the worst. The reason for this result may be that while the on average long answers of ChatGPT might lead some users into thinking the LLMs explain the case in detail, the overall explanation for the therapy of primary head and neck cancer was not convincing to the reviewing head and neck oncologists. ChatGPT 4.0 had the overall better performance with a better rate of concordance with the MDT, probably due to the ability to access more data than ChatGPT 3.5, as demonstrated in a few studies (8, 10, 15).

There have been a few studies investigating the use of ChatGPT in the field of medical and surgical oncology due to the ability to rapidly extract and deal with a large quantity of information (7, 9, 20). ChatGPT is often reduced to an auxiliary tool or assistant rather than a replacement of the MDT in most studies. One of these studies investigated decision making with ChatGPT for ten consecutive patients with primary breast cancer. This study compared the treatment recommendations of ChatGPT with the MDT and found similarities in 7 out of 10 cases (9). Compared to the results for primary head and neck cancer, a similar Cohen’s κ and grades for summarization, explanation and clinical recommendation were found, while our study additionally analyzed the number of therapy options as a potential indicator of the quality of ChatGPT. For primary breast cancer a high reference rate for surgery of 80% was found, similar to the rate of 90% (for ChatGPT 3.5) seen for head and neck cancer in this study. For the use in otolaryngology a recent publication discovered in a clinical case series of laryngology and oncological cases a performance of decision making of 60-68% (15). That study used ChatGPT 4.0 as a tool in the clinical setting focusing on diagnostic examinations and the general clinical workflow without addressing the specific needs and situation of the MDT. Another study also investigated the use of ChatGPT for breast cancer and used prompts in German, leading to a lower concordance with mostly general answers, probably since the literature and studies and therefore the source material of ChatGPT is predominantly in English and may impair the access of ChatGPT (12). In a recent study by Benary et al., the fictional cases of 10 patients with advanced cancer with genetic alterations were submitted to 4 different LLMs and one physician to identify personalized treatment options. The number of treatment options, precision, recall, F1 score of LLMs compared with human experts, recognizability, and usefulness of recommendations were graded, but the results did not reach the quality of human experts (21). The number of therapy options recommended by LLMs was large, similar to the results of our study for head and neck cancer, while the authors summarized that LLMs mainly support idea generation and cannot be used in the current oncological routine. This finding is very similar to the high number of therapy options recommended by ChatGPT for head and neck cancer in our study. ChatGPT’s ability to summarize a vast number of therapy options is at the moment just informative, since the participants of the MDT would potentially also be able to name the therapy options, but name only the therapy option with the best prognosis or the one that is most suitable to the patient’s current situation. The ability to name the therapy options could be used for teaching purposes and to enable the patient to make an informed decision but is currently hindered by a lack of information provided on the prognosis of the patient based on the suggested therapy. Maybe for this reason, ChatGPT explicitly states in most responses that it is not programmed as a medical bot and the literature reveals that there has been no prior oncological training of ChatGPT (7, 20) Therefore other research groups investigated the use of a clinical decision support system based on Bayesian networks (BN) for laryngeal cancer (LC) as a prototype with over 1,000 variables (22–24). The TNM classification was the main classifier for the therapeutic recommendations of the Bayesian Network, compared to the ability of ChatGPT 4.0 in this study to address the comorbidities, extent of the tumor and in the case of ChatGPT 4.0 some of the latest studies, including the quality of life of a patient. Limitations of this study of Bayesian Networks include the limited access of other physicians to the software, the integration of only laryngeal carcinoma and a general lack of data on immunotherapy due to insufficient data. On the other hand, ChatGPT accessed and cited recent studies, including immunotherapy, while also referencing some rarely used therapy options, as way of thinking outside the box, similar to the conclusion of Benary et al., 2023 (21).

The major benefits of using AI in the setting of the tumor board tackle some of the biggest challenges of the physical MDT, while most of these positive aspects are connected to the digital interface of it. Like in a virtual tumor board, the participants of the AI-guided MDT may benefit from the exceptional accessibility of ChatGPT without geographical boundaries. While the MDT at the hospital where this study was performed, hosts physical MDTs, an AI guided MDT may boost the number of participating physicians by not limiting them to a specific place. Currently ChatGPT is free of charge in version 3.5, which makes it an accessible tool for physicians in developing countries, where resources in terms of oncological knowledge and surgical expertise are limited to major hospitals. Another potential use is the ability to summarize large datasets of information and extract key content from records of patients to facilitate a more informed decision making process as suggested by some studies (20). There was no direct comparison of the time spent in a physical MDT versus the MDT with ChatGPT, but ChatGPT answered the prompts in this study in an exceptionally fast way, taking mere seconds. On of the other hand, ChatGPT in it’s current version has a few limitations. For ChatGPT 3.5 the source information used for the answer is missing. Every medical professional in the MDT has to discuss the therapy options with other specialists and has to base his opinion on scientific ground (2, 3). Without naming the source of information there can be no discussion based on the answers of ChatGPT. A limitation of this study is that even though this study analyzes one of the larger cohorts of patients compared to the literature for breast cancer, only a small patient cohort (n =20) was analyzed, impacting the study results due to inherent patient heterogeneity. To make up for this, only consecutive primary cases of HNSCC were included in this proof-of-concept study. Future studies may need to investigate ChatGPT-driven MDT decisions in a more homogeneous patient group, focusing on only one specific location of HNSCC. Another drawback is that in this study the MDT of only one institution was investigated, whereas other MDTs might decide cases differently due to historical or resource/local reasons, or simply because of different physicians being present (3). Another limitation is that ChatGPT is based on a large language model and will access information that is based on the prompt (12, 14). Four different prompts were used initially, but only one prompt, identical to the way a case would have been presented at the MDT was used for this study to directly compare the results to the MDT. This is a limitation, that might have influenced the results of this study, since the prompt can have a significant impact on the answers of ChatGPT as seen in other studies. ChatGPT will, at least in the current version, not ask for additional information, whereas the participants of the MDT will demand more information if the available information is insufficient, or the decision-making is difficult. This was also confirmed in other studies (11, 21). In addition, it remains to be elucidated whether a large dataset of clinical data from the MDT may be able to train ChatGPT or a different AI tool to get more tailored recommendations. Within the clinical context of a MDT, ChatGPT 4.0 was significantly superior to ChatGPT 3.5 in terms of transparency of the information, citing sources, including a clinical study on quality of life after laryngeal surgery. For one patient with extensive tumor growth, ChatGPT 3.5 chose a different treatment than the MDT, while ChatGPT 4.0 was able to recommend the right treatment. The most important limitation and one of the reasons for the results in this study is that ChatGPT is not built to think independently, producing texts based on public documents and databases (5). It is therefore not able to make individual decisions, thereby failing to tailor the therapy to the patient’s current situation and needs. The treatment plan of the MDT combines the clinical knowledge of the patient and the results of recent studies, to optimize a patient’s prognosis, while also balancing potential downsides and risks of the therapy (14, 21). When one thinks of the three potential use cases of ChatGPT in the future, a fully AI-guided MDT, an assistant to the MDT, or a validation method, ChatGPT, even in its most advanced version (ChatGPT 4.0) is currently merely able to assist the MDT. The reason for this is, that the ChatGPT was able to reproduce text from online databases to name a vast number of therapy options, but doesn’t address the scientific clinical studies in this study that lead the MDT to recommend a specific therapy for a patient, while in theory ChatGPT 4.0 is able to produce convincing answers in terms of summarization, explanation and clinical recommendation. An ideal AI-guided MDT would be able to evaluate the most recent oncological studies and compare the results to the standard operating procedure to enable a more up to date and faster decision-making process. In the closer future an AI-validation by LLMs could already assess the decisions of the MDT and comment on the scientific basis, with the task of tailoring the treatment to the patient still in the hands of the MDT. Nonetheless it will remain the MDTs participants task to carefully evaluate the recommendations based on their own clinical knowledge and knowledge of the patient’s situation.

## 5 Conclusions

In the current version ChatGPT is able to list the various treatment options for primary head and neck cancer. Even though there was no prior oncological training, ChatGPT, especially in Version 4.0 reached exceptionally high scores for explanation and clinical recommendation by two independent reviewers. The limitations of ChatGPT, including a lack of personalized treatment planning, the recommendation of incorrect treatment guidelines and in the case of ChatGPT 3.5 a lack of information on the source material, currently allows ChatGPT to merely assist the MDT for head and neck cancer cases.

## Data availability statement

The original contributions presented in the study are included in the article/**Supplementary Material**. Further inquiries can be directed to the corresponding author.

## Ethics statement

Ethical approval was not required for the study involving humans in accordance with the local legislation and institutional requirements. Written informed consent to participate in this study was not required from the participants or the participants’ legal guardians/next of kin in accordance with the national legislation and the institutional requirements.

## Author contributions

BS: Conceptualization, Formal analysis, Funding acquisition, Methodology, Resources, Validation, Visualization, Writing – original draft, Writing – review & editing. ToH: Formal analysis, Methodology, Writing – review & editing. SP: Conceptualization, Investigation, Resources, Supervision, Writing – review & editing. FS: Data curation, Formal analysis, Methodology, Writing – review & editing. CH: Data curation, Formal analysis, Validation, Writing – review & editing. TiH: Conceptualization, Methodology, Supervision, Writing – review & editing. BW: Conceptualization, Methodology, Supervision, Validation, Writing – review & editing. MW: Conceptualization, Resources, Supervision, Validation, Writing – original draft.
