# Supplementary figures and images for: Assessing the role of advanced artificial intelligence as a tool in multidisciplinary tumor board decision-making for primary head and neck cancer cases
Source: Front Oncol. 2024 May 24;14:1353031. doi: 10.3389/fonc.2024.1353031 (PMC11157509; doi:10.3389/fonc.2024.1353031)

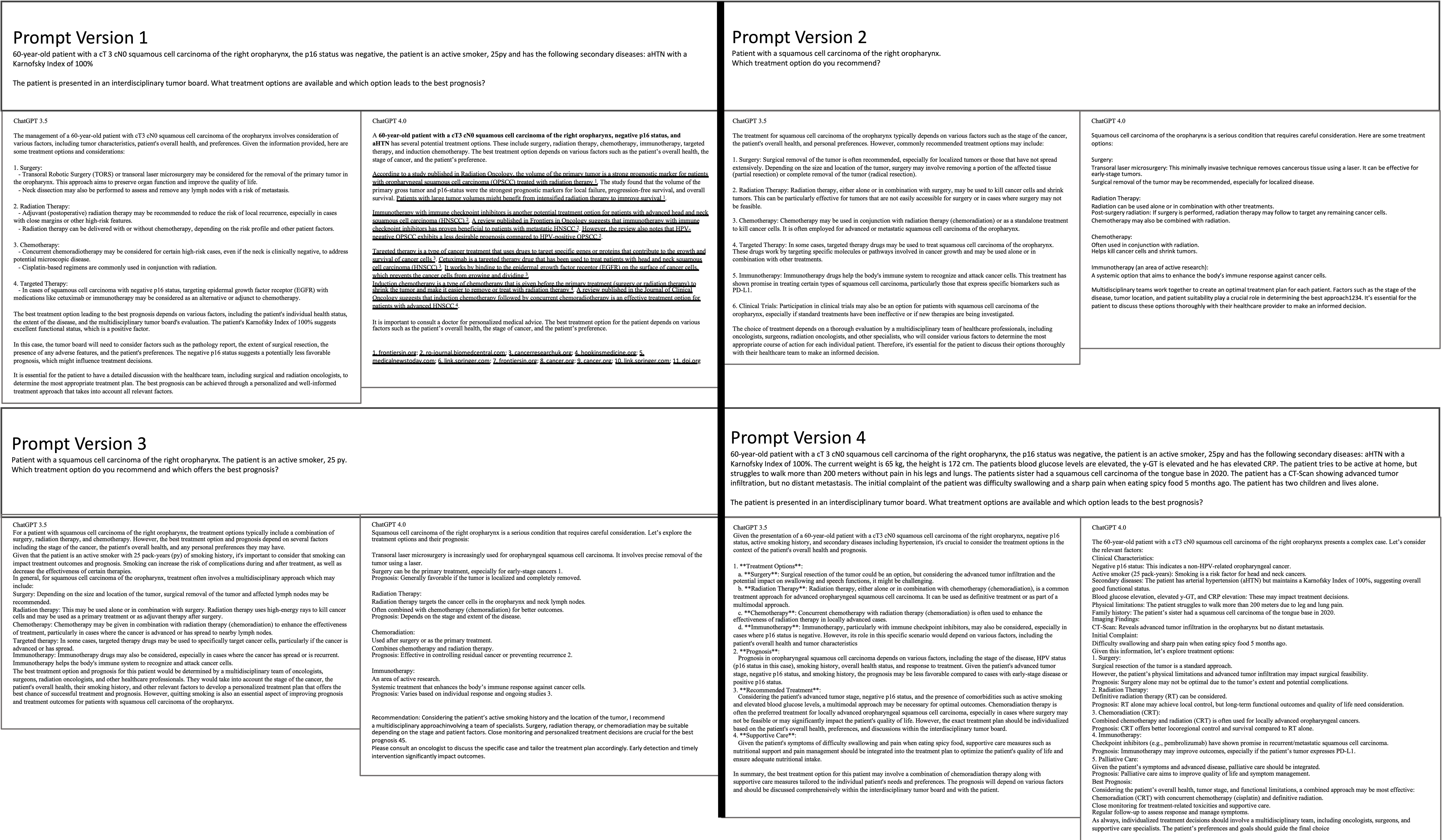

Supplement: Supplementary file 1 [file Image_1.jpg]
